# How Pronatalist Framing of Family Policies in Iran and Türkiye Have Unwittingly Entrenched the Medicalization of Childbirth

**DOI:** 10.34172/ijhpm.9968

**Published:** 2026-07-28

**Authors:** Sevil Hakimi, AbouAli Vedadhir

**Affiliations:** ^1^Department of Midwifery, Faculty of Health Science, Ege University, Izmir, Türkiye.; ^2^Department of Anthropology, Faculty of Social Sciences, University of Tehran, Tehran, Iran.; ^3^Department of Population Health Sciences, Bristol Medical School, University of Bristol, Bristol, UK.

## Dear Editor,

 An expanding array of low-fertility societies across the Global North and Global South have recently turned toward renewed family explicitly pronatalist policy agendas,^1^ a shift that prominently includes Iran and Türkiye. Today, these two countries are amongst top 20 countries with the highest populationwith almost parallel populations. Both countries confront the enduring challenge of low fertility, a demographic dilemma that has galvanized a new wave of pronatalist and family policy initiatives. Key demographic, fertility, and maternity care indicators for Iran and Türkiye are summarized in Table.

**Table:** Comparative Demographic Indicators for Iran and Türkiye

**Demographic Indicator**	**Iran**	**Türkiye**
Population^2^	91.5 million	85.5 million
TFR (2024)^3,4^	1.44	1.48
Cesarean rate (%) (2022)	56.11	60.5^5^

Abbreviation: TFR, total fertility rate.

 Over the past two decades, hence, both countries have adopted a constellation of pronatalist family policies. In line with these policies, a set of initiatives to boost population growth by promoting marriage and childbearing, promoting natural vaginal delivery (NVD), reducing rates of the caesarean section (CS) and restricting free contraception, prenatal screening and abortion services has been devised, introduced and implemented in both countries.^1,6^

 Both Iran and Türkiye have implemented policies aimed at reducing high CS rates. In Iran, interventions included in-service midwifery training, confidential audit-and-feedback mechanisms for obstetricians, and financial incentives promoting NVD.^7^ Similarly, Türkiye introduced the Physician Unit Performance Coefficient policy in 2013, which financially rewards physicians with lower primary CS rates while reducing payments for those with higher rates.^8^

 In short, neither country has succeeded in raising fertility to replacement level, while both first-birth and overall CS rates have remained persistently high or continued to increase since the 2000s, positioning Iran and Türkiye as global outliers in CS prevalence (Table).

 Indeed, the CS has steadily become the preferred mode of childbirth in both Iran and Türkiye, irrespective of its substantial burdens, ranging from prolonged maternal recovery to heightened risks in subsequent pregnancies, women fall disproportionately on what may be called *“the middlecut mothers,”* who bear the brunt of this medicalized intervention.^2^ Additionally unnecessary CS may further diminish fertility intentions by amplifying fears of repeat surgery, escalating economic burdens, or reinforcing perceptions of heightened health risks.^9^

## Framing Question

 Despite these initiatives and policy architectures in countries such as Iran and Türkiye, a deeper question endures: How do policies, medical authority, and ideas about risk quietly work together to promote having more children, while at the same time bringing pregnancy more under medical control?

 Across the globe, several countries have implemented pronatalist policies over the years; however, many of these initiatives have achieved only limited success. For example, countries such as Hungary and the Czech Republic introduced various pronatalist measures, yet these policies did not fully succeed in substantially increasing fertility rates.^10,11^

 Pronatalist policies often treat childbirth as a matter of national priority. This can lead to institutionalization by encouraging or requiring hospital births to ensure safety which naturally increases medical oversight. In some regions, high birth targets can lead to “scheduled” births (C-sections) to manage hospital flow.

 Within pronatalist settings, CS may be implicitly or explicitly encouraged as a means of guaranteeing timely, predictable, and medically “safe” births, especially in contexts where fetal loss or adverse outcomes are sensitive. Additionally, financial incentives, institutional protocols, and clinician risk aversion, often amplified under pronatalist discourse, further normalize, and routinize CS as a default intervention.^2^

 Many of family policies with pronatalist aims suffer from profound knowledge gaps, compounded by marked different types of paradoxes (eg, information/epistemic; implementation; signification) and policy incompatibilities and asymmetries. They are further weakened by persistent mis-targeting, inadequate inter-sectoral coordination, recurrent administrative mismanagement, and, above all, the absence of a contextualized, comprehensive, and genuinely holistic policy vision.^1^

 The persistent paradoxes and incompatibilities within the pronatalist agendas stem from their foundational reliance on medicalized, centralized, and top-down implementation. These policies are operationalized through a hegemonic, physician-centred care model and a hospital-centric obstetric infrastructure. This top-down agenda systematically marginalizes alternative paradigmsand fails to empower women in their sexual and reproductive choices and instead reinforces a hegemonic, technocratic, and biomedical model of birth.^12^ As a result, pronatalist policies can unintentionally contribute to rising CS rates by reinforcing a biomedical model of childbirth.

## Can We Fix This?

 Health, family and population policies need to be aligned so that sexual and reproductive health and rights are fully supported. Both governments should prioritize midwife-led care for low-risk pregnancies, as this approach reduces unnecessary interventions while protecting both mothers and newborns.

 Although midwifery-led care is well established and widely integrated into maternity services in many high-income countries, its adoption in low- and middle-income settings remains relatively recent and still evolving. Countries such as the Netherlands, Sweden, and New Zealand—where midwifery-led continuity models are strongly embedded within maternity systems—have also maintained comparatively lower CS rates than many other high-income settings.^13^

 In parallel, strengthening preparation classes and antenatal education for mothers is essential, as these programs can help reduce fear of NVD and promote informed decision-making. In addition, obstetrics and gynecology residency curricula should be revised to place greater emphasis on supporting and managing NVD, strengthening residents’ competencies and confidence in physiological birth practices. This change is not merely technical; it requires reforming education systems so that midwives are central to maternity care. At the same time, institutional, financial, and structural barriers must be addressed. Midwives should be supported by a strong legal framework that empowers them with clear authority and decision-making capacity.

 Financial and monitoring systems must also be recalibrated to reward the quality and continuity of maternal care. Financial penalties alone are unlikely to solve the problem; instead, they may incentivize the misclassification of CS as emergency cases, leading to inaccurate reporting and distorted national statistics. Public health campaigns are also essential to highlight the clinical safety, physiological benefits, and broader sociocultural value of NVD for mothers, children, and society at large.

 Cultural norms can normalize CS when surgical birth becomes socially associated with safety, modernity, higher socioeconomic status, convenience, or reduced suffering. Fear of labor pain and childbirth complications in the pronatalist settings, these dynamics may become even stronger because pregnancy and childbirth are framed as highly sensitive national or demographic concerns. Under such conditions, both institutions and families may become increasingly risk-averse, unintentionally reinforcing interventionist childbirth practices. To ameliorate these attitudes, interventions must go beyond clinical regulations, addressing the broader social culture surrounding childbirth.

 Potential strategies include: expanding midwife-led continuity-of-care models; strengthening antenatal education focused on physiological birth; providing effective labor pain management and emotional support; increasing public visibility of positive vaginal birth experiences; training healthcare professionals in respectful maternity care; reducing fear-based counselling and unnecessary risk framing; promoting shared decision-making rather than physician-dominated decisions; reforming hospital environments that routinely medicalize low-risk birth; using public health campaigns to normalize physiological childbirth; and ensuring women receive balanced, evidence-based information about both CS and vaginal birth.

## Conclusion

 In conclusion, the persistence of exceptionally high CS rates in Iran and Türkiye underscores a profound disjunction between pronatalist ambitions and the lived realities of maternal health. As long as the structural, institutional, cultural, and medical forces that drive the medicalization of childbirth remain unaddressed, efforts to increase fertility are unlikely to succeed and may, paradoxically, intensify the very demographic challenges they aim to resolve.

 Bridging this divide requires a genuine realignment of population policies with reproductive health and rights strategies. This entails the empowerment of midwives, the promotion of evidence-based and low-intervention models of childbirth, and the development of maternity care systems that are truly centered on women’s needs, experiences, and choices. Crucially, population governance must move beyond narrowly instrumental rationality approaches to fertility and instead engage with the ethical, sociocultural, and embodied dimensions of reproduction.

## Disclosure of artificial intelligence (AI) use

 The authors used ChatGPT (GPT-5.2, OpenAI) to support English language editing during the preparation of this letter to editor.

## Ethical issues

 This study relied exclusively on publicly available open data and was therefore exempt from institutional review board approval.

## Conflicts of interest

 Authors declare that they have no conflicts of interest.
